# Prognostic value of immune-related genes in clear cell renal cell carcinoma

**DOI:** 10.18632/aging.102548

**Published:** 2019-12-10

**Authors:** Bangbei Wan, Bo Liu, Yuan Huang, Gang Yu, Cai Lv

**Affiliations:** 1 Department of Urology, Central South University Xiangya School of Medicine Affiliated Haikou Hospital, Haikou, Hainan 570208, China; 2 Laboratory of Developmental Cell Biology and Disease, School of Ophthalmology and Optometry and Eye Hospital, Wenzhou Medical University, Wenzhou 325003, China; 3 Department of Neurology, Central South University Xiangya School of Medicine Affiliated Haikou Hospital, Haikou, Hainan 570208, China

**Keywords:** immune, ccRCC, TCGA database, prognosis, immune-related genes

## Abstract

Clear cell renal cell carcinoma (ccRCC) is the most common pathological subtype of renal cell carcinoma, and immune-related genes (IRGs) are key contributors to its development. In this study, the gene expression profiles and clinical data of ccRCC patients were downloaded from The Cancer Genome Atlas database and the cBioPortal database, respectively. IRGs were obtained from the ImmPort database. We analyzed the expression of IRGs in ccRCC, and discovered 681 that were differentially expressed between ccRCC and normal kidney tissues. Univariate Cox regression analysis was used to identify prognostic differentially expressed IRGs (PDEIRGs). Using Lasso regression and multivariate Cox regression analyses, we detected seven optimal PDEIRGs (*PLAU*, *ISG15*, *IRF9*, *ARG2*, *RNASE2*, *SEMA3G* and *UCN*) and used them to construct a risk model to predict the prognosis of ccRCC patients. This model accurately stratified patients with different survival outcomes and precisely identified patients with different mutation burdens. Our findings suggest the seven PDEIRGs identified in this study are valuable prognostic predictors in ccRCC patients. These genes could be used to investigate the developmental mechanisms of ccRCC and to design individualized treatments for ccRCC patients.

## INTRODUCTION

Renal cell carcinoma is one of the most lethal cancer types in the urinary system, and its morbidity has been increasing year after year [1]. Clear cell renal cell carcinoma (ccRCC) is the most common subtype of renal cell carcinoma, accounting for approximately 70-85% of cases [2]. Although therapeutic treatments for ccRCC have improved, the mortality rate is still high, especially for patients with advanced/metastatic ccRCC [3]. Hence, to improve the prognosis of ccRCC patients, it is important to identify biomarkers for the prognostic prediction and treatment of ccRCC.

In recent years, immunotherapy has become an important method of enhancing the survival outcomes of ccRCC patients [4, 5]. Certain immune checkpoint molecules (for instance, programmed death 1 [PD-1]) are popular targets of immunotherapy, and immune checkpoint inhibitors have been reported to attenuate tumor growth mainly by reducing the immune escape of cancer cells [6, 7]. However, some patients are insensitive to immune checkpoint inhibitors. Therefore, it is important to identify high-performance biomarkers that predict patients’ sensitivity to immunotherapy so that individualized treatments for ccRCC can be implemented.

Previous studies have suggested that immune-related genes (IRGs) are associated not only with the response to immunotherapy, but also with the prognosis of ccRCC patients [8, 9]. Chen et al. reported that *HHLA2* was significantly overexpressed in ccRCC tissues and was associated with a poor prognosis [10]. Kammerer-Jacquet et al. found that PD-L1 expression was higher in ccRCC tissues than in paired renal cortex tissues, and that the prognosis was worse in patients expressing PD-L1 than in those with undetectable PD-L1 expression [11].

Although several studies have investigated the association of IRGs with the prognosis of ccRCC patients, the majority of these studies focused on the function of a single gene. Few studies have employed expression profile datasets from high-throughput sequencing to examine the relationships of multiple immune genes with the prognosis of ccRCC. Therefore, in this study, we developed a reliable prognostic model of ccRCC using IRGs, and investigated the clinical utility of this model in ccRCC patients.

## RESULTS

### Expression of IRGs in ccRCC

**Figure 1:**
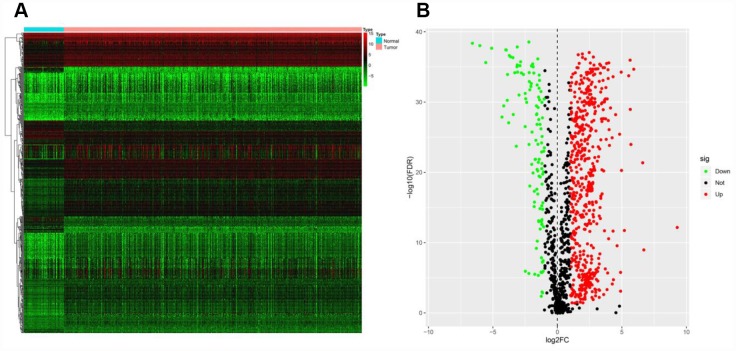
**Expression of IRGs in the two sample groups.** (**A**) Heat map of IRGs; the green to red spectrum indicates low to high gene expression. (**B**) Volcano plot of IRGs; the green dots represent downregulated IRGs, the red dots represent upregulated IRGs and the black dots represent IRGs that were not significantly differentially expressed.

The mRNA levels of 2498 IRGs in ccRCC (n = 539) and normal kidney tissues (n = 72) in The Cancer Genome Atlas (TCGA) were examined, and these values were compared through the Wilcoxon signed-rank test. This analysis revealed 681 differentially expressed IRGs (DEIRGs), including 565 genes that were upregulated and 116 genes that were downregulated in ccRCC tissues compared with normal kidney tissues (false-discovery rate [FDR] < 0.05, |log2 fold-change [FC]| > 1) (Figure 1).

### Identification of prognostic DEIRGs

**Figure 2:**
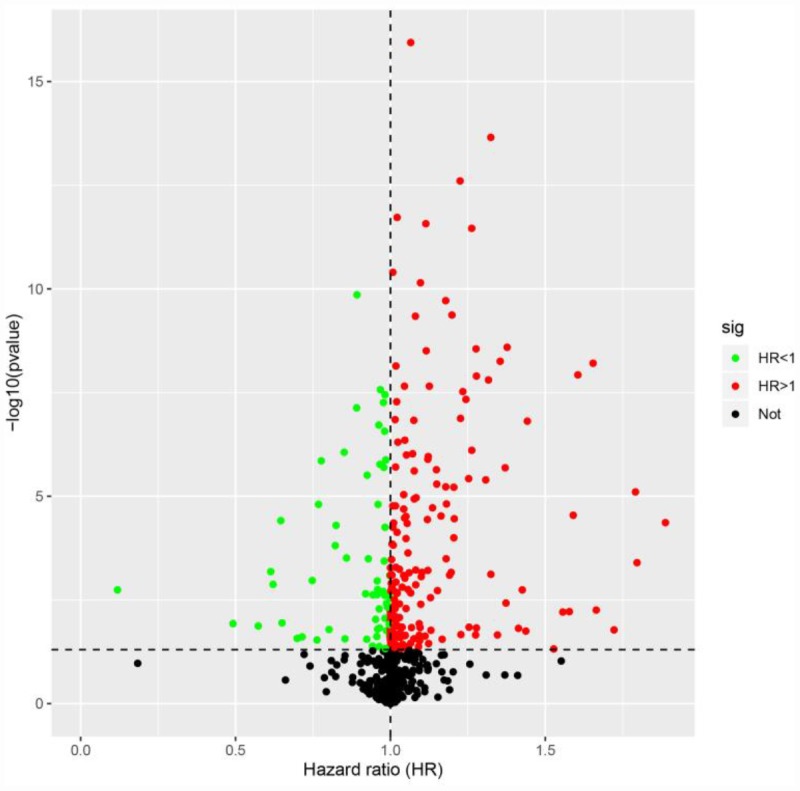
**Identification of PDEIRGs through univariate Cox regression analysis.** The red dots represent DEIRGs with hazard ratios > 1 (*p* < 0.05), the green dots represent DEIRGs with hazard ratios < 1 (*p* < 0.05) and the black dots represent DEIRGs that were not associated with prognosis (*p* > 0.05).

To identify possible prognostic DEIRGs (PDEIRGs), we performed a univariate Cox regression analysis of the expression of each DEIRG in the entire TCGA cohort. In total, 263 DEIRGs were found to be significantly associated with the overall survival (OS) of ccRCC patients (*p* < 0.05) (Figure 2).

### Construction of a transcription factor regulatory network

**Figure 3:**
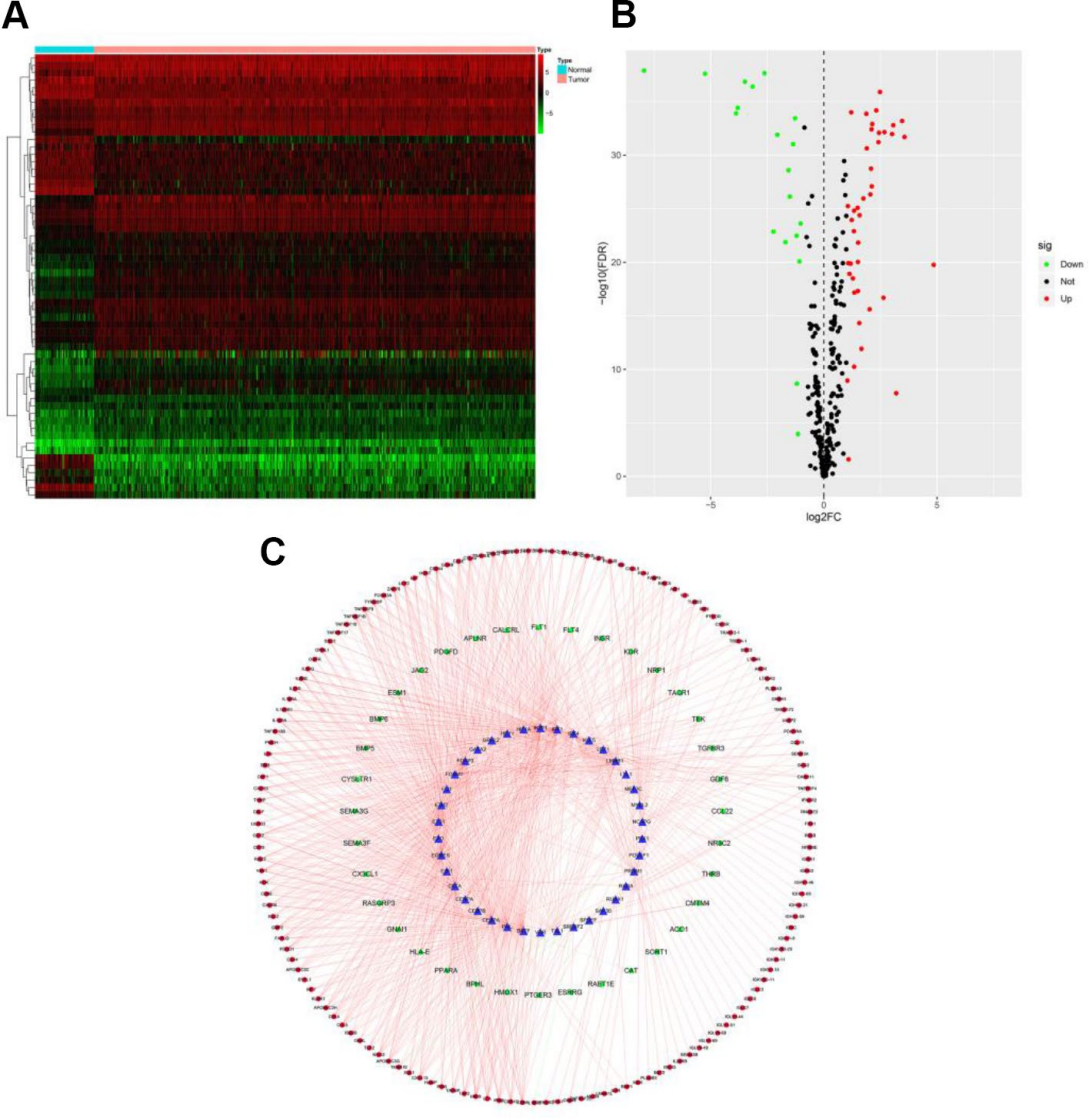
**TF-based regulatory network.** (**A**) Heat map of differentially expressed TFs; the green to red spectrum indicates low to high TF expression. (**B**) Volcano plot of TFs; the green dots represent downregulated TFs, the red dots represent upregulated TFs and the black dots represent TFs that were not significantly differentially expressed. (**C**) Regulatory network of TFs and PDEIRGs; the green nodes represent PDEIRGs with hazard ratios < 1 (*p* < 0.05), the red nodes represent PDEIRGs with hazard ratios > 1 (*p* < 0.05), the blue nodes represent TFs that correlated with the PDEIRGs in terms of their mRNA levels (correlation coefficient > 0.4 and *p* < 0.05), the green lines indicate negative regulatory relationships and the red lines indicate positive regulatory relationships.

To determine the possible mechanisms behind the dysregulation of PDEIRG expression in ccRCC, we analyzed the correlation between cancer transcription factor (TF) and PDEIRG expression. First, we examined the mRNA levels of TFs in ccRCC (n = 539) and normal kidney tissues (n = 72), and identified 60 TFs (FDR < 0.05, |log2 FC| > 1) that were significantly differentially expressed between the two tissue types (Figure 3A and 3B). Next, we analyzed the correlations between the mRNA levels of the 60 TFs and the PDEIRGs, using a correlation coefficient > 0.4 and a *p*-value < 0.05 as the cut-off values. Among the 60 TFs, 38 were prominently associated with the aberrant expression of PDEIRGs (*p* < 0.05). To better explain the regulatory relationships, we constructed a TF-based regulatory network, as displayed in Figure 3C.

### Training cohort to identify prognostic genes for inclusion in the risk model

**Figure 4:**
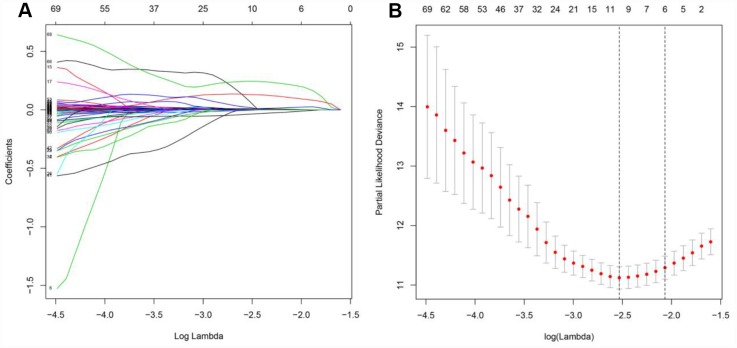
**Further analysis of the PDEIRGs in the training cohort.** (**A** and **B**) PDEIRGs selected through Lasso regression.

**Figure 5:**
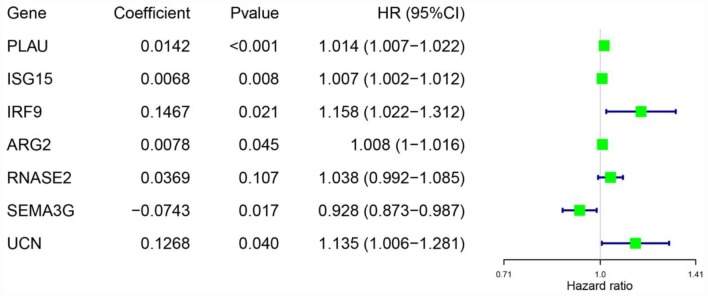
**Risk genes in the prognostic risk model.**

Considering the impact of the PDEIRGs on the OS of patients, we further screened the PDEIRGs to construct a Cox regression hazards model. For this analysis, we used 266 of the 530 patients as a training cohort. First, to avoid overfitting the model, we used Lasso regression to delete PDEIRGs that correlated highly with one another. We thus obtained 12 candidate PDEIRGs (Figure 4A and 4B), and further analyzed them via multivariate Cox proportional hazards regression analysis (with forward selection and backward selection). Ultimately, we obtained seven optimal PDEIRGs (risk genes) for inclusion in the prognostic risk model: *PLAU*, *ISG15*, *IRF9*, *ARG2*, *RNASE2*, *SEMA3G* and *UCN*. Among these genes, *PLAU*, *ISG15*, *IRF9*, *ARG2*, *RNASE2* and *UCN* were identified as high-risk genes (predicting a poor prognosis), while *SEMA3G* was identified as a low-risk gene (serving as a protective factor) in terms of the OS of patients (Figure 5).

### Construction of the prognostic risk model in the training cohort

To investigate the significance of the risk genes in predicting the prognosis of ccRCC patients, we used the mRNA levels and estimated regression coefficients of the risk genes to calculate a risk score for each patient. The computational formula was as follows:

Training cohort risk score = (0.0142 × expression of *PLAU*) + (0.0068 × expression of *ISG15*) + (0.1467 × expression of *IRF9*) + (0.0078 × expression of *ARG2*) + (0.0369 × expression of *RNASE2*) + (-0.0743 × expression of *SEMA3G*) + (0.1268 × expression of *UCN*).

**Figure 6:**
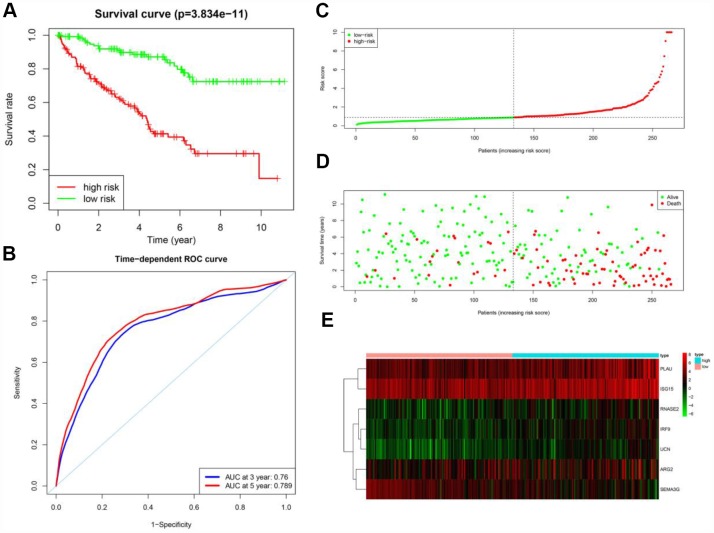
**Prognostic analysis of the training cohort.** (**A**) Kaplan-Meier curve analysis of the high-risk and low-risk groups. (**B**) Time-dependent ROC curve analysis of the prognostic model. (**C**) Risk score distribution of patients in the prognostic model. (**D**) Survival status scatter plots for patients in the prognostic model. (**E**) Expression patterns of risk genes in the prognostic model.

According to the median risk score, the patients in the training cohort were sorted into a high-risk group (n = 133) and a low-risk group (n = 133). To determine the prognostic difference between the high-risk and low-risk groups, we created a Kaplan-Meier curve based on the log-rank test. The prognosis was worse in the high-risk group than in the low-risk group (*p* < 0.05) (Figure 6A). The OS rates at three years and five years for the high-risk group in the training cohort were 63.1% and 41.4%, respectively, while the corresponding rates for the low-risk group were 90.8% and 87.1%, respectively. We then used time-dependent receiver operating characteristic (ROC) curves to examine the predictive accuracy of the model for OS at three years and five years. The area under the ROC (AUC) values for the prognostic model were 0.760 at three years and 0.789 at five years (Figure 6B). We then ranked the risk scores of the patients in the training cohort and analyzed their distribution (Figure 6C). The survival status of each patient in the training cohort is marked on the dot plot in Figure 6D.

A heat map was generated to depict the expression patterns of the risk genes in the two prognostic groups (Figure 6E). In patients with high risk scores in the training cohort, the six high-risk genes (*PLAU*, *ISG15*, *IRF9*, *ARG2*, *RNASE2* and *UCN*) were upregulated, while the protective gene (*SEMA3G*) was downregulated. In patients with low risk scores, these risk genes displayed the opposite expression pattern.

### Verification of the performance of the prognostic model

To verify the accuracy of the prognostic risk model, we used it to analyze the testing cohort (the remaining 264 patients from the 530 total) and the entire TCGA cohort. First, we used the seven risk genes (*PLAU*, *ISG15*, *IRF9*, *ARG2*, *RNASE2*, *SEMA3G* and *UCN*) to calculate the risk score of each patient in the testing cohort and the entire TCGA cohort. The patients in each cohort were then classified into two groups based on the median risk score of the training cohort. In the testing cohort, 148 patients were categorized as high-risk and 116 were categorized as low-risk. In the entire TCGA cohort, 290 patients were classified as high-risk and 240 were classified as low-risk.

**Figure 7:**
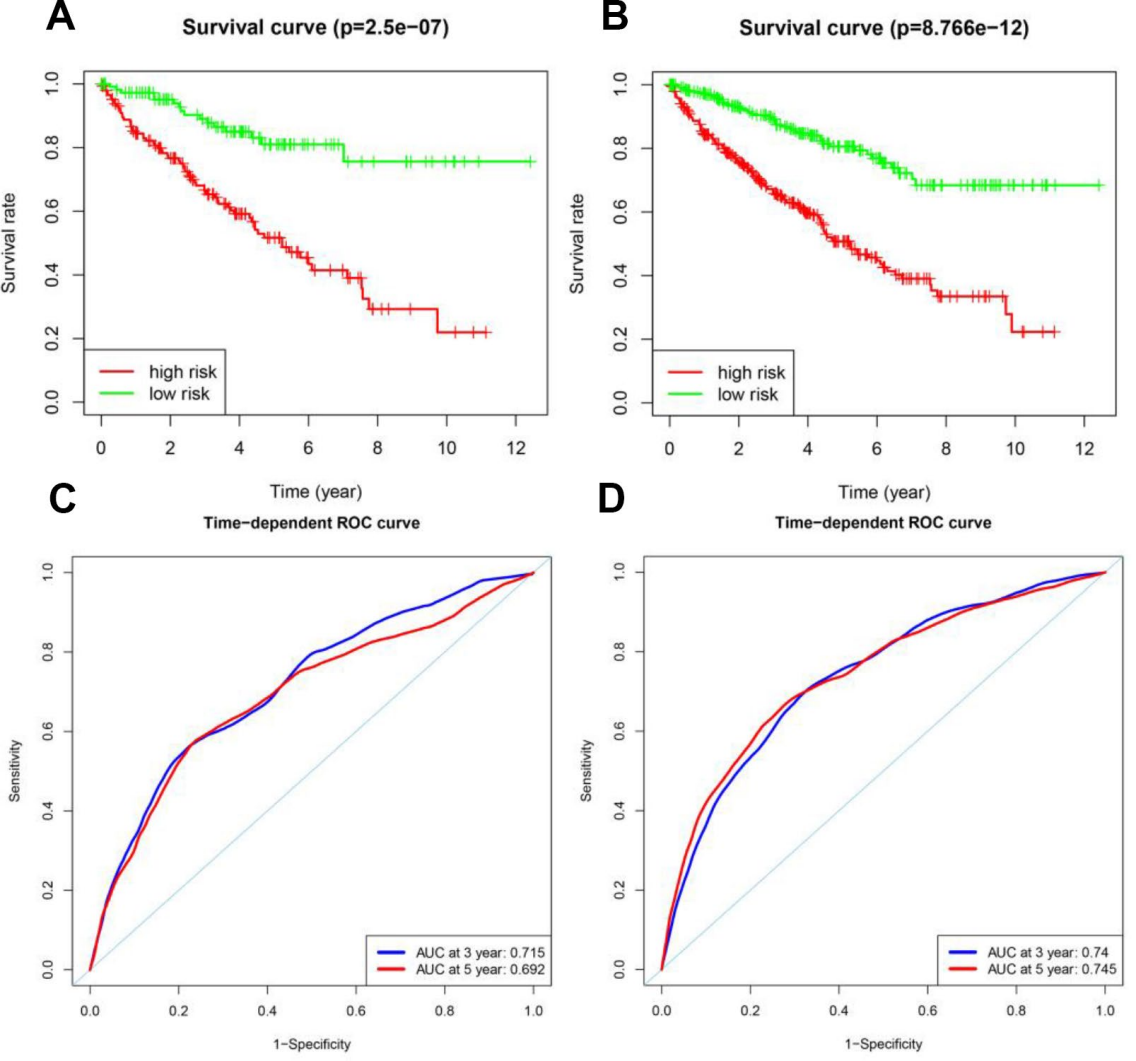
**Prognostic analyses of the testing cohort and the entire TCGA cohort.** (**A**) Kaplan-Meier curve analysis of high-risk and low-risk patients in the testing cohort. (**B**) Kaplan-Meier curve analysis of high-risk and low-risk patients in the entire TCGA cohort. (**C**) Time-dependent ROC curve analysis of the testing cohort. (**D**) Time-dependent ROC curve analysis of the entire TCGA cohort.

Next, Kaplan-Meier survival analysis was used to determine the prognostic differences between the high-risk and low-risk groups. The Kaplan-Meier survival curves differed significantly between the two risk groups in both the testing cohort and the entire TCGA cohort (*p* < 0.05) (Figure 7A and 7B); throughout the follow-up time, the survival rate was higher in low-risk patients than in high-risk patients. In the testing cohort, the survival rates at three and five years in the high-risk group were 66.2% and 51.7%, respectively, while the corresponding rates in the low-risk group were 89.0% and 81.0%, respectively. In the entire TCGA cohort, the survival rates at three and five years in the high-risk group were 66.3% and 50.8%, respectively, while the corresponding rates in the low-risk group were 89.2% and 80.6%, respectively. ROC analyses were performed for the testing cohort and the entire TCGA cohort at three and five years. In the testing cohort, the AUCs at three and five years were 0.715 and 0.692, respectively. In the entire TCGA cohort, the AUCs at three and five years were 0.740 and 0.745, respectively (Figure 7C and 7D).

**Figure 8:**
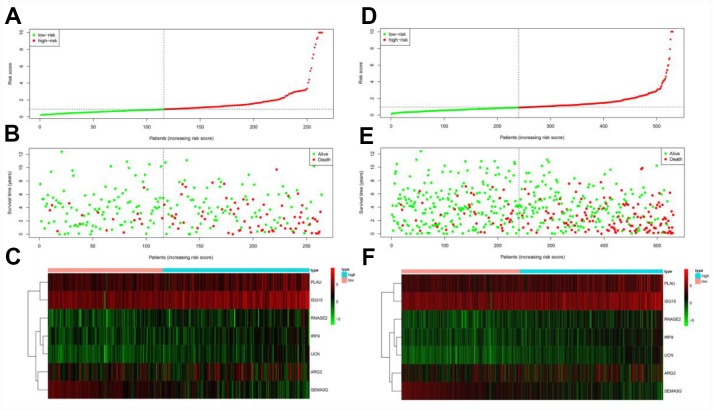
**Prognostic analyses of high-risk and low-risk patients in the testing cohort and the entire TCGA cohort.** (**A**) Risk score distribution of patients in the testing cohort. (**B**) Survival status scatter plots of patients in the testing cohort. (**C**) Expression patterns of risk genes in the testing cohort. (**D**) Risk score distribution of patients in the entire TCGA cohort. (**E**) Survival status scatter plots of patients in the entire TCGA cohort. (**F**) Expression patterns of risk genes in the entire TCGA cohort.

The risk score distribution, survival status and risk gene expression in the testing cohort and the entire TCGA cohort are displayed in Figure 8A–8F. Similar to the results in the training cohort, protective gene levels were higher and risk gene levels were lower in the low-risk group than in the high-risk group. These results indicated that our prognostic risk model is capable of precisely predicting the prognosis of ccRCC patients.

### Independent prognostic value of the risk model in the entire TCGA cohort

Next, we performed univariate and multivariate Cox regression analyses to assess whether the risk score generated by our model was independent from other clinical parameters (age, gender, histological grade and pathological stage) as a prognostic factor for ccRCC. The univariate analysis indicated that the variables of age, histological grade, pathological stage and risk score were associated with the prognosis of ccRCC patients. The multivariate analysis revealed that the risk score was independently associated with OS in the entire TCGA cohort (*p* < 0.05) (Table 1). These results demonstrated that the prognostic risk model can be used independently to predict the prognosis of ccRCC patients. However, the three clinical variables (age, histological grade and pathological stage) were also found to be significant prognostic factors in the multivariate analysis (*p* < 0.05).

**Table 1:** Univariate and multivariate Cox regression analyses of the entire TCGA cohort.

**Variables**	**Univariate analysis**		**Multivariate analysis**	
	**HR (95% CI)**	***p*-value**	**HR (95% CI)**	***p*-value**
	Overall survival			
Risk score (from risk model)	1.21 (1.16-1.26)	1.10E-18	1.14 (1.08-1.20)	4.23E-07
Age	1.02 (1.01-1.04)	2.16E-05	1.03 (1.01-1.04)	5.89E-06
Gender	0.96 (0.70-1.31)	0.797		
Histological grade	2.27 (1.85-2.78)	3.00E-15	1.42 (1.13-1.79)	0.002
Pathological stage	1.87 (1.64-2.13)	1.10E-20	1.62 (1.39-1.89)	3.38E-10

HR, hazard ratio; CI, confidence interval

**Figure 9:**
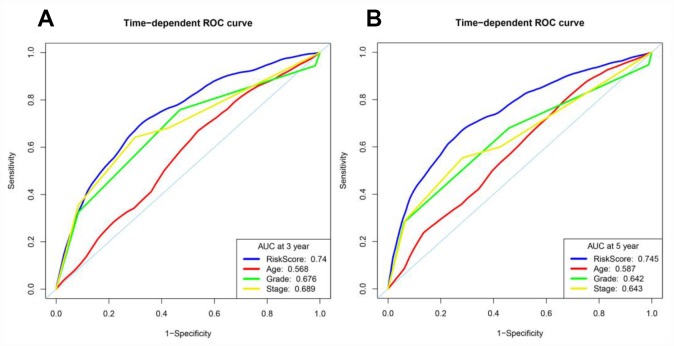
**Time-dependent ROC curve analyses of different variables in the entire TCGA cohort at three and five years.** (**A**) AUC at three years. (**B**) AUC at five years.

We then assessed whether the risk score from our model was more accurate than the other clinical parameters (age, histological grade and pathological stage) in predicting OS at three and five years. Indeed, the risk score was more accurate than the other clinical parameters: the AUCs at three years for age, histological grade and pathological stage were 0.568, 0.676 and 0.689, respectively (Figure 9A), and the corresponding values at five years were 0.587, 0.642 and 0.643, respectively (Figure 9B).

**Figure 10:**
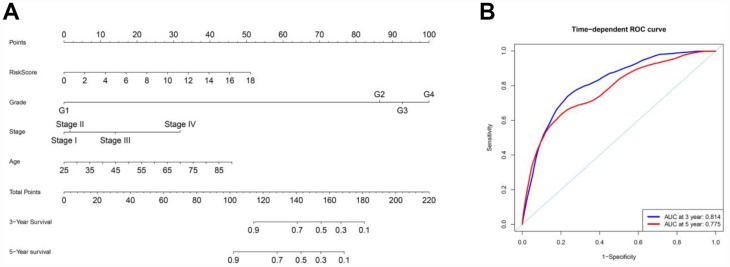
**Nomogram and ROC curves for the prediction of prognosis at three and five years in the entire TCGA cohort.** (**A**) Nomogram for OS. (**B**) ROC curves for OS.

To better predict the prognosis of ccRCC patients at three and five years post-surgery, we constructed a new nomogram from the variables associated with OS (age, histological grade, pathological stage and risk score) (Figure 10A). ROC curve analysis was used to evaluate the accuracy of the nomogram. The nomogram could accurately predict OS at three and five years post-surgery, with AUCs of 0.814 and 0.775, respectively (Figure 10B).

### Clinical utility of the prognostic risk model

**Figure 11:**
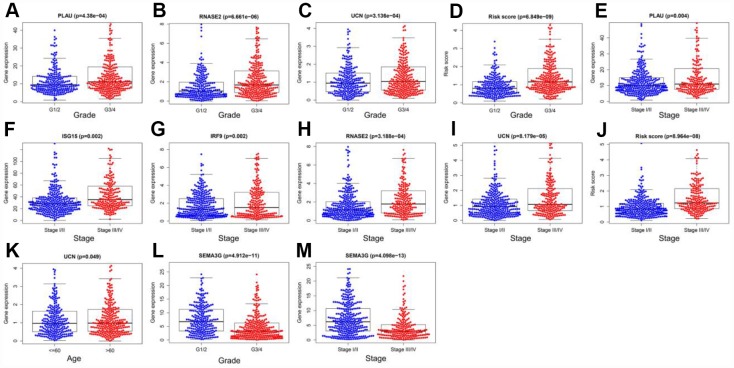
**Relationships of the variables in the model with the clinical characteristics of patients in the entire TCGA cohort.** (**A**) *PLAU* expression and histological grade. (**B**) *RNASE2* expression and histological grade. (**C**) *UCN* expression and histological grade. (**D**) Risk score and histological grade. (**E**) *PLAU* expression and pathological stage. (**F**) *ISG15* expression and pathological stage. (**G**) *IRF9* expression and pathological stage. (**H**) *RNASE2* expression and pathological stage. (**I**) *UCN* expression and pathological stage. (**J**) Risk score and pathological stage. (**K**) *UCN* expression and age. (**L**) *SEMA3G* expression and histological grade. (**M**) *SEMA3G* expression and pathological stage.

To examine the ability of our model to predict the progression of ccRCC, we analyzed the relationships between the risk factors from our model (the risk score and risk genes) and the clinical variables (age, gender, histological grade and pathological stage) in the entire TCGA cohort. As the values of certain factors increased (*PLAU*, *RNASE2* and *UCN* levels and the risk score), the histological grade of ccRCC patients increased (all *p* < 0.05) (Figure 11A–11D), and as the values of other factors increased (*PLAU*, *ISG15*, *IRF9*, *RNASE2* and *UCN* levels and the risk score), the pathological stage of ccRCC patients increased (all *p* < 0.05) (Figure 11E–11J). The expression of *UCN* was higher in patients > 60 years old than in those ≤ 60 years old (*p* < 0.05) (Figure 11K). In contrast, as *SEMA3G* expression increased, the values of two clinical variables (histological grade and pathological stage) decreased (both *p* < 0.05) (Figure 11L and 11M) (Table 2). These results demonstrated that the dysregulation of immune-related risk gene expression is associated with the development of ccRCC.

**Table 2:** Relationships between model variables and clinical variables in the entire TCGA cohort.

**Variables**	**Age (>60/≤60)**	**Gender (male/female)**	**Histological grade (I&II/III&IV)**	**Pathological stage (I&II/III&IV)**
	**t (*p*)**	**t (*p*)**	**t (*p*)**	**t (*p*)**
*PLAU*	-0.066 (0.948)	0.119 (0.905)	-3.543 (4.38E-04)	-2.925 (0.004)
*ISG15*	-1.271 (0.204)	-0.556 (0.578)	-1.775 (0.077)	-3.102 (0.002)
*IRF9*	0.345 (0.730)	1.657 (0.098)	-1.437 (0.151)	-3.06 (0.002)
*ARG2*	-0.546 (0.585)	0.932 (0.352)	-1.907 (0.057)	-1.176 (0.241)
*RNASE2*	-0.726 (0.468)	-1.482 (0.139)	-4.567 (6.661E-06)	-3.65 (3.188E-04)
*SEMA3G*	1.514 (0.131)	1.838 (0.067)	6.734 (4.912E-11)	7.447 (4.098E-13)
*UCN*	-1.973 (0.049)	0.312 (0.755)	-3.631 (3.136E-04)	-3.995 (8.179E-05)
Risk score	-1.636 (0.102)	-0.271 (0.786)	-5.946 (6.849E-09)	-5.521 (8.964E-08)

t: t value from Student’s t test; *p*: *p*-value from Student’s t test.

**Figure 12:**
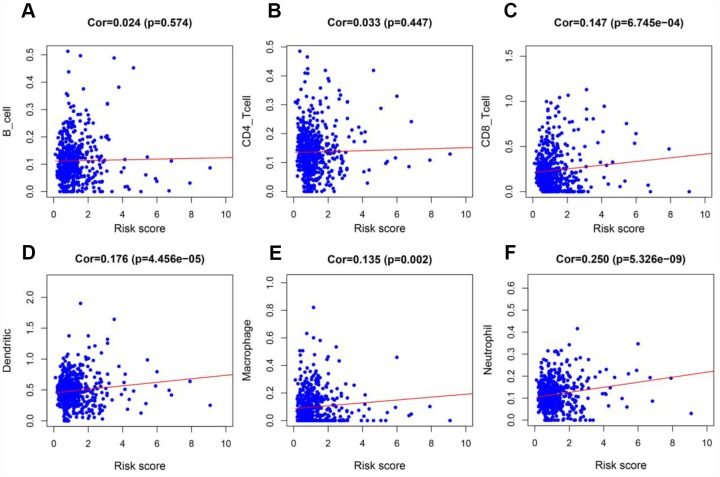
**Analysis of the correlation between the risk score and immune cell infiltration in the entire TCGA cohort.** (**A**) B cells. (**B**) CD4+ T cells. (**C**) CD8+ T cells. (**D**) Dendritic cells. (**E**) Macrophages. (**F**) Neutrophils.

To determine whether our model could reflect the status of the tumor immune microenvironment in patients, we analyzed the correlation between the risk score and immune cell infiltration in the entire TCGA cohort. As the risk score increased, the content of immune cells (CD8+ T cells, neutrophils, macrophages and dendritic cells) in ccRCC tissues also increased (*p* < 0.05) (Figure 12A–12F).

**Figure 13:**
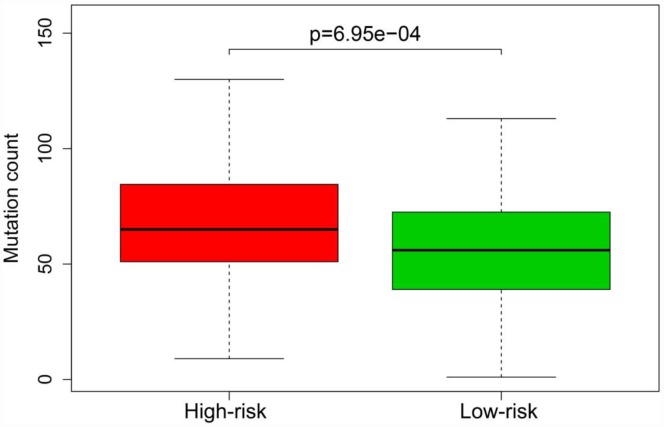
**Mutation burden of patients in the high-risk and low-risk groups of the entire TCGA cohort.**

Previous studies have indicated that the tumor mutation burden is significantly associated with the clinical effectiveness of immunotherapy [12]. To assess whether our model could stratify patients with different sensitivities to immunotherapy, we compared the mutation counts of the high-risk and low-risk groups in the entire TCGA cohort. The mutation count was greater in the high-risk group than in the low-risk group (*p* < 0.05) (Figure 13).

## DISCUSSION

The activation of the immune system is a decisive factor during cancer initiation and progression [13, 14], as immune cells can kill cancer cells by up- or downregulating IRGs at certain immune checkpoints [15, 16]. However, some cancer cells can elude this destruction by mimicking the IRG expression patterns of healthy cells [17, 18]; this process is called immune escape. Hence, IRG expression may be an important predictor of the progression and prognosis of ccRCC. In this work, we identified IRGs associated with prognosis, and used them to construct a dependable model to predict OS in ccRCC patients.

First, we analyzed the expression of 2498 IRGs in ccRCC, and obtained 681 DEIRGs, including 565 upregulated and 116 downregulated genes. We then performed a univariate Cox regression analysis to examine the relationship of these 681 DEIRGs with the prognosis of ccRCC patients, and found that the expression of 263 DEIRGs correlated with OS. These results revealed that IRGs are vital contributors to the prognosis of ccRCC patients. To explore the potential molecular mechanisms behind the aberrant expression of these PDEIRGs, we constructed a TF regulatory network, and found that 38 TFs were associated with the expression of the PDEIRGs. These results illustrated that TFs determined the impact of the PDEIRGs on patients’ OS. This TF regulatory network will provide the foundation for future studies on the developmental mechanisms of ccRCC.

Next, we examined the value of these PDEIRGs for the prognostic stratification of patients. We identified seven PDEIRGs of interest (*PLAU*, *ISG15*, *IRF9*, *ARG2*, *RNASE2*, *SEMA3G* and *UCN*) through a combination of Lasso regression and Cox regression analyses, and used them to construct a Cox regression hazards model. We then further analyzed the reliability and stability of the model and validated it. Our results indicated that the model could accurately discriminate between patients with different survival outcomes. Univariate and multivariate Cox regression analyses demonstrated that our model could independently predict the prognosis of ccRCC patients. A nomogram analysis indicated that combining the model with other clinical characteristics (age, histological grade and pathological stage) increased its accuracy in predicting ccRCC prognosis. Thus, our model can be used to identify ccRCC patients at high risk for death, enabling early interventions to improve the prognosis of patients in clinical work.

We also analyzed the relationships of the factors in our model with certain clinical variables (age, gender, histological grade and pathological stage). We found that various factors in the model (such as *PLAU*, *ISG15*, *IRF9*, *RNASE2* and *UCN* expression) correlated positively with the progression of ccRCC. Thus, our model exhibited high clinical applicability in predicting the development of ccRCC.

Previous studies have demonstrated that immune infiltration is an important determinant of the therapeutic responsiveness and prognosis of ccRCC [19, 20]. Şenbabaoğlu et al. found that reduced contents of certain immune cells (such as Th17 cells) were associated with the progression of ccRCC and a poor patient prognosis [21]. George et al. reported that the disease-free survival of patients with high CD8+ T-cell densities could be significantly prolonged through the use of sunitinib therapy [22]. Therefore, we also analyzed the relationship between the risk score from our model and immune cell infiltration, and found that the risk score correlated positively with the infiltration of certain immune cells (CD8+ T cells, neutrophils, macrophages and dendritic cells). These results also verified the reliability of the model in predicting the prognosis of ccRCC.

Previous reports have indicated that the tumor mutation burden correlates positively with the occurrence of a neoantigen that can significantly enhance the effects of immunotherapy [12, 23, 24]. Thus, we examined whether our model reflected patients’ tumor mutation burden, and found that the mutation burden was higher in the high-risk group than in the low-risk group. These results suggested that the model can be used to distinguish patients with different sensitivities to immunotherapy, making individualized treatment strategies a possibility.

The value of IRG models for predicting the prognosis of cancer patients has been described in previous studies. Song et al. used nine IRGs to develop a Cox regression model to predict the prognosis of patients with lung adenocarcinoma, and found that the model could accurately stratify patients with different survival outcomes. The authors also compared the tumor mutation burdens of patients in the high-risk and low-risk groups, and found that the mutation burden was greater in the high-risk group [25]. Wang et al. constructed a prognostic risk model using 15 IRGs in renal papillary cell carcinoma, and found that the model could independently distinguish patients with different risks of death [26]. Lin et al. employed a Cox regression model of IRGs for the prognostic stratification of patients with papillary thyroid cancer, and found that the IRG model could discriminate patients with high and low risks of death [27]. Our work differed from these previous studies in several ways. Firstly, we focused on the IRG expression pattern in ccRCC. Secondly, we used multiple algorithms (including univariate Cox, multivariate Cox and Lasso regression) to identify IRGs for inclusion in our model, so our study was more reliable than the others. Thirdly, the IRGs in our model did not overlap with those in the previous models. Importantly, our model was better than the previous models at predicting immune cell infiltration, the mutation burden and the progression of ccRCC.

Inevitably, our study also had some shortcomings. Firstly, we used data from public databases that were not validated in prospective clinical trials. Additionally, the mechanisms whereby the identified IRGs impact the development of ccRCC require further investigation with *in vivo* and *in vitro* experiments.

In summary, we constructed a risk model using seven IRGs that precisely predicted the prognosis of patients with ccRCC. The risk score generated by this model can be used as an independent prognostic marker to distinguish patients with different survival outcomes. Additionally, the model can stratify patients with different mutation burdens and help to predict the sensitivity of patients to immunotherapy. However, further experiments are required to verify the findings of this study.

## MATERIALS AND METHODS

### Databases

The 2498 IRGs were obtained from the ImmPort database (https://www.immport.org/home). The 539 ccRCC patients’ data, including transcriptomic data, mutation data and clinical information, were downloaded from the TCGA portal (https://portal.gdc.cancer.gov/) and the cBio Cancer Genomics portal (https://www.cbioportal.org/) [28, 29]. All data were processed with R software (https://www.r-project.org/). We matched patients’ transcriptomic data and clinical information according to their ID numbers, and we removed patients if their ID numbers did not match. We thus obtained data from 530 patients with complete gene expression profiles and OS information (Table 3). Immune infiltrate data from the ccRCC patients was obtained from the Cistrome project (http://www.cistrome.org/) [30], which contains the abundances of six types of tumor-infiltrating immune cells (B cells, CD4+ T cells, CD8+ T cells, neutrophils, macrophages and dendritic cells). Data on TFs associated with cancer were also collected from the Cistrome project.

**Table 3:** Clinical information from the 530 ccRCC patients.

**Clinical parameters**	**Variable**	**n (total = 530)**	**Percentages (%)**
Age (years)	≤ 60	259	48.9%
	> 60	271	51.1%
Gender	Female	186	35.1%
	Male	344	64.9%
Histological grade	G1	14	2.6%
	G2	227	42.8%
	G3	206	38.9%
	G4	75	14.2%
	GX	5	1.0%
	Unknown	3	0.5%
Pathological stage	Stage I	265	50.0%
	Stage II	57	10.8%
	Stage III	123	23.2%
	Stage IV	82	15.5%
	Unknown	3	0.5%
Survival status	Dead	173	32.6%
	Alive	357	67.4%

### Identification of DEIRGs

The Wilcoxon signed-rank test was used to screen DEIRGs based on the following cut-off values: FDR < 0.05 and |log2 FC| > 1.

### Experimental model construction

The 530 patients were randomly divided into two cohorts: a training cohort (n = 266) and a testing cohort (n = 264) (Table 4). The training cohort was used to construct the Cox regression hazards model, and the testing cohort was used to test the performance of the model. Initially, univariate Cox analysis was used to identify possible PDEIRGs. Next, Lasso regression was used to select potential risk genes and eliminate genes that would overfit the model. Finally, we used Cox proportional hazards regression to construct a prognostic risk model.

**Table 4:** Grouping of the ccRCC patients

**Clinical parameter**	**Variable**	**Training cohort**	**Testing cohort**	**Entire TCGA cohort**
Survival status	Dead	89 (16.8%)	84 (15.8%)	173 (32.6%)
	Alive	177 (33.4%)	180 (34.0%)	357 (67.4%)

### Risk score calculation

To calculate the risk score for each patient, we used the regression coefficients from the multivariate Cox regression model to weight the expression values of the selected genes. The following computational formula was used for this analysis:

$$Risk\;score\;(patient)=\overset{n}{\underset{i=1}{\sum }}coefficien\;t\;(gene\;i)\;expression\;value\;of\;(gene\;i)$$

Here, ‘gene_i_’ is the i^th^ selected gene, and ‘coefficient (gene_i_)’ is the estimated regression coefficient of gene_i_ from the Cox proportional hazards regression analysis. The risk model was used to measure the prognostic risk of each patient with ccRCC. The median risk score of the training cohort was used as the cut-off value to divide all the ccRCC patients into two groups: the high-risk group and the low-risk group. A high risk score indicates a poor prognosis for ccRCC patients.

### Statistical analyses

R software was used to perform all statistical analyses, and *p* < 0.05 was considered statistically significant. The rank correlation among the different variables was assessed with the Pearson correlation coefficient test. Differences between variables were assessed with independent t-tests. Kaplan-Meier curves and log-rank tests were used to analyze the survival data, and univariate Cox regression analysis was used to identify factors affecting the survival of patients diagnosed with ccRCC. Multivariate Cox regression analysis was used to identify independent prognostic factors. Time-dependent ROC analysis was used to evaluate the accuracy of the prognostic prediction model. An AUC > 0.60 was regarded as acceptable for predictions, and an AUC > 0.75 was deemed to have excellent predictive value [31, 32].
